# Supplementary material for: HIVprotI: an integrated web based platform for prediction and design of HIV proteins inhibitors
Source: J Cheminform. 2018 Mar 9;10:12. doi: 10.1186/s13321-018-0266-y (PMC5845081; doi:10.1186/s13321-018-0266-y)
Supplement: Supplementary file 2 — Additional file 2. Source code of HIVProtI web server. [file 13321_2018_266_MOESM2_ESM.zip › HIVprotI_Source-code/hivprotI/weka-3-6-6/changelogs/CHANGELOG-3-5-5.html]

Weka-3-5-5 Changelog


# Weka-3-5-5 Changelog

Designed for use with Apache Ant.

---

2007-01-26 02:12 fracpete

```
updated version number
```

- core/version.txt (1.4)

2007-01-26 02:11 fracpete

```
added more files to the release (from wekadocs module):
documentation.html/css, weka.gif, weka.ico
```

- build.xml (1.36)

2007-01-25 23:38 fracpete

```
updated copyright
```

- gui/GUIChooser.html (1.27)
- gui/Main.html (1.8)

2007-01-25 22:18 fracpete

```
added new schemes
```

- gui/GenericObjectEditor.props (1.116)

2007-01-25 21:49 fracpete

```
fixed line break in -z option (for Sourcable classifiers)
```

- classifiers/Evaluation.html (1.73)

2007-01-25 21:09 fracpete

```
fixed comments
```

- filters/unsupervised/attribute/PartitionedMultiFilter.html (1.2)

2007-01-24 20:52 fracpete

```
added @see Javadoc tags for filters being used (Discretize and, if used as well, NominalToBinary)
```

- attributeSelection/CfsSubsetEval.html (1.24)
- attributeSelection/ChiSquaredAttributeEval.html (1.12)
- attributeSelection/ConsistencySubsetEval.html (1.14)
- attributeSelection/GainRatioAttributeEval.html (1.20)
- attributeSelection/InfoGainAttributeEval.html (1.18)
- attributeSelection/SymmetricalUncertAttributeEval.html (1.19)
- attributeSelection/SymmetricalUncertAttributeSetEval.html (1.6)

2007-01-24 03:37 fracpete

```
fixed PerformanceComparator for accuracy measure (like correlation coefficient: the bigger the better)
```

- classifiers/meta/GridSearch.html (1.3)

2007-01-23 23:33 fracpete

```
initial import
```

- filters/unsupervised/attribute/NumericCleaner.html (1.1)

2007-01-23 03:54 fracpete

```
added the BibTeX tags and updated the Javadoc
```

- classifiers/trees/BFTree.html (1.2)
- classifiers/trees/SimpleCart.html (1.2)

2007-01-21 20:52 fracpete

```
fixed typo in globalInto() method
```

- filters/unsupervised/attribute/TimeSeriesTranslate.html (1.7)

2007-01-21 04:46 fracpete

```
fixed typo in error message
```

- gui/treevisualizer/TreeVisualizer.html (1.11)

2007-01-14 21:26 fracpete

```
fixed technical information from MISC to MASTERSTHESIS
```

- classifiers/mi/TLD.html (1.5)
- classifiers/mi/TLDSimple.html (1.5)

2007-01-14 21:25 fracpete

```
implements now the weka.core.TechnicalInformationHandler interface
transferred all the references from Javadoc to the getTechnicalInformation() method
fixed Javadoc in general
```

- core/Optimization.html (1.7)

2007-01-10 01:18 fracpete

```
batch filtering didn't set the correct dataset for instances of the second dataset before getting passed through the filter, resulting in a dataset with class index -1, even though the class index was set via the '-c' option. this can result in a "headers don't match" exception (e.g., AttributeSelection filter in combination with PCA)
```

- filters/Filter.html (1.34)

2007-01-08 23:30 fracpete

```
added merge functionality to commandline: weka.core.Instances merge <file1> <file2>
uses now DataSource to read any data format instead of only ARFF format
```

- core/Instances.html (1.67)

2007-01-08 23:29 fracpete

```
the mergeInstance method produced corrupt results or NullPointerException in case the datasets had different number of attributes
thanks to Scott Davies
```

- core/SparseInstance.html (1.17)

2006-12-19 20:22 fracpete

```
fixed NullPointerExceptions if no file extension was provided explicitely
```

- gui/ConverterFileChooser.html (1.6)
